# Child Schooling in Ethiopia: The Role of Maternal Autonomy

**DOI:** 10.1371/journal.pone.0167639

**Published:** 2016-12-12

**Authors:** Tesfaye Alemayehu Gebremedhin, Itismita Mohanty

**Affiliations:** 1 Faculty of Business, Government and Law, University of Canberra, Canberra, Australia; 2 Centre for Research and Action in Public Health, Health Research Institute, University of Canberra, Canberra, Australia; TNO, NETHERLANDS

## Abstract

This paper examines the effects of maternal autonomy on child schooling outcomes in Ethiopia using a nationally representative Ethiopian Demographic and Health survey for 2011. The empirical strategy uses a Hurdle Negative Binomial Regression model to estimate years of schooling. An ordered probit model is also estimated to examine age grade distortion using a trichotomous dependent variable that captures three states of child schooling. The large sample size and the range of questions available in this dataset allow us to explore the influence of individual and household level social, economic and cultural factors on child schooling. The analysis finds statistically significant effects of maternal autonomy variables on child schooling in Ethiopia. The roles of maternal autonomy and other household-level factors on child schooling are important issues in Ethiopia, where health and education outcomes are poor for large segments of the population.

## I. Introduction

Investment in schooling is an important means of improving economic growth and well-being. Nonetheless, educational attainment continues to be low in many developing countries. Moreover, wide gender disparities are observed, with female children typically receiving less education than their male counterparts [1–6]. Because schooling investments are typically made at the household level, poor households must make decisions about the optimum allocation of scarce resources towards child schooling. Hence, in addition to economic factors, a range of child specific characteristics, such as the child’s gender, birth order, and the number and sex composition of their siblings, also become important considerations in schooling decisions [7, 8].

This paper analyses the importance of women’s autonomy on child schooling decisions at the household level. Improvements in maternal socio-economic status have been strongly linked to better demographic outcomes and improved child welfare [9–12]. For instance, ‘….Increased female autonomy has been shown to confer.....benefits like long-term reduction in fertility, higher child survival rates and allocation of household resources in favour of children’ [13]. Women are typically the primary caregivers for children, which puts them in a unique position to influence the well-being of children by investing in their nutrition, health and education. DasGupta and Mani (2015) [14] established that women are often the more altruistic members of the family, favouring joint household consumption goods that would promote the welfare of the whole family; this is in contrast to men, who show a strong preference for private consumption goods. There is evidence that women with financial autonomy and increased decision-making power allocate more resources to their children, leading to better health and education outcomes [15, 16]. In rural Mexico, for example, educational grants offered to poor mothers raise the school enrolment rate of children [17]. Rangel (2006) [18] indicated that a shift in the balance of decision power (within households), through changing alimony rights to favour women, leads to redistribution of resources towards the schooling of first-born girls in Brazil. Women’s autonomy and the resultant changes in the household’s labour supply decisions have also been associated with greater productivity and economic growth [19]. Despite these benefits resulting from improving the socio-economic status of women, women continue to have little household decision-making authority in many developing countries.

The Ethiopian constitution accords equal rights to women and men in various spheres of life, including marriage, property rights and inheritance [20]. Laws that uphold the rights of women have been promulgated, including the prohibition of female genital mutilation (FGM), abolition of polygamy and lifting of the minimum age of marriage for girls from 15- to 18-years old. However, Ethiopia’s informal institutions remain unequal and early marriages, high fertility rates, low educational attainment and entrenched patriarchal attitudes continue to undermine women’s autonomy [21]. FGM is still prevalent, with an incidence of over 70 per cent [22], and polygamy continues in practice [23]. The central government’s ability to enforce laws is limited because the federal system gives full sovereignty to states, allowing them to continue upholding older laws that are not favourable to women. Moreover, women lack the political representation and clout required to pressure the government to enforce gender equality laws [23].

In this paper, we examine the effect of maternal autonomy on child schooling outcomes in Ethiopia using a nationally representative dataset from the Demographic and Health Survey (DHS). The role of maternal autonomy on schooling has not been studied in the Ethiopian context but is of considerable importance in a region in which female socio-economic status is considered to be poor. Specifically, we analyse whether indicators of maternal economic and social autonomy play a role in improving children’s schooling outcomes (after controlling for several other variables that affect schooling).

Our focus on Ethiopia is motivated by several factors. Ethiopia is among the poorest countries in the world, with a GDP per capita (2005 PPP $) of $979 in 2011, which is considerably lower than the Sub-Saharan African average of $2,094 [24]. Second, the Ethiopian government has invested heavily in education through programs such as the Education Sector Development Programmes (ESDP). The goal of this program has been to increase access to education (at all levels) and improve the quality of education, particularly for girls, rural residents and other disadvantaged groups [25, 26]. This led to a higher share of the budget being allocated to the education sector and a sharp increase in the number of schools, institutions and teachers; as a consequence, it also led to a significant increase in access to education at all levels [26]. The net enrolment rate at the primary level, for example, increased from 40.3 per cent in 2000 to 86.5 per cent by 2011 [27]. Despite these achievements in boosting enrolments (especially at the primary level), schooling outcomes remain poor in Ethiopia. For instance, completion rates remain quite low and show a worsening trend. The primary school completion rate, which was approximately 61 per cent in 2000, dropped to 41 per cent by 2010 [27]. Moreover, approximately 2.3 million primary school age children in Ethiopia were out-of-school in 2010. Secondary and tertiary education are both similarly dogged by problems of retention and progression, in addition to having low rates of enrolment. The persistence of poor schooling outcomes in the face of rapid public investments in education suggests that it is important to examine the role of household-level factors and existing social systems and practices, with a particular focus on the role of maternal autonomy and ethnicity in influencing schooling decisions.

Our analysis takes into account school enrolment, the number of years of schooling and information on those children who may have dropped out of school or who are over-aged for their grade. By linking schooling attainment to age-for-grade attainment or relative grade attainment [28, 29], we use a measure of schooling outcomes that is appropriate for a developing country such as Ethiopia, where child labour is an issue and there are cases of combining schooling with work. This measure allows us to take into account age-grade distortions that would not be possible with more conventional measures such as current enrolment or actual grade attainment.

Our analytical approach to examine the links between maternal autonomy and child schooling considers several maternal autonomy variables as reported by the child’s mother. The empirical strategy in the paper first uses a Hurdle model to account for the two-stage decision-making process in child schooling: the decision to enrol in school and the decision to continue in school conditional on enrolment. Second, the paper uses an ordered probit model with a trichotomous dependent variable to capture the age for grade or the relative grade attainment of the child through three schooling states as a function of a range of maternal autonomy, individual, household, social and economic characteristics. Both models take into account the concern that some children in the sample are currently continuing in school and so their years of schooling are right censored.

The remainder of the paper is organized as follows. Section II will discuss the data sources and methodology used in the study. The list of exogenous variables used in the estimation model is also described in this section. Section III will present a discussion of the results. And section IV will provide a summary and some concluding remarks.

## II. Data and Methods

The data used in this analysis come from the 2010–11 *Ethiopian Demographic and Health Survey* (EDHS). This dataset is rich, unique, and nationally representative. Our analysis uses household level information on parental education, employment status, occupational status, sibling characteristics and mother’s autonomy and socioeconomic status. The sample was selected using a stratified, two-stage cluster design. Enumeration Areas (EAs) are the sampling units at the first stage. Kebeles, which are the lowest administrative units in Ethiopian administrative structure, were the basis on which the EAs were selected. Administratively, regions in Ethiopia are divided into zones, and zones are divided into administrative units called *weredas*. Each *wereda* is further subdivided into the lowest administrative unit, called a *kebele*.

The official starting age for school in Ethiopia is 7 years. The education system ‘consists of an eight-year primary education cycle, which is itself divided into a basic education cycle covering grades 1–4, and a general primary cycle covering grades 5–8, followed by two years of general secondary education (grades 9–10), and two years of preparatory secondary education (grades 11–12)’ [30]. The official starting age for general secondary education is 15 years and for preparatory secondary education is 17 years [30]. A child enrolled in school at the official starting age (for all levels and who progresses without failing) will complete primary education (including both basic and general primary) at age 14 and secondary education (including general and preparatory secondary) at age 18. Following these standards and considering the possibilities of late enrolments and dropouts, we have focused on the 8–20-year-old school-going age group, which in our sample consists of 11,949 children.

### Methodology

This paper examines the effects of household socio-economic characteristics, and the role of maternal autonomy, in particular, on child schooling outcomes in Ethiopia. The *Ethiopian Demographic and Health Survey* (EDHS) provides information on two important dimensions of schooling: (i) whether the child is ever enrolled in school and (ii) the years of schooling completed.

However, enrolment by itself is not a complete indicator of overall child schooling outcomes; a significant proportion– 33.6 per cent–of children in the sample have never been enrolled. Further, current enrolment and schooling status is likely to be biased in those cases in which the child is lagging behind the appropriate grade for their age. For example, if the starting age for schooling is 7, we expect a child progressing at the appropriate age to have completed 4 years of schooling by age 11. However, using information on the child’s current enrolment status tends to overestimate schooling outcomes for those children who are currently enrolled but who may have had long absences in the past.

Furthermore, by excluding children who are not currently enrolled, we lose valuable information on those children who may have dropped out after some schooling. Putting these children in the same category as those who have never attended school may lead to biased estimates because these children may have dropped out of school after gaining basic literacy and numeracy skills. Also the descriptive statistics show that the data are skewed towards primary schooling, with 95 per cent of the children ever enrolled having primary complete or less as their highest level of educational attainment.

The focus of this paper is primarily to analyse the completed years of schooling using a Hurdle Model and to analyse age appropriate/relative years of schooling using an Ordered probit model. Both models are estimated using household level sampling weights available with EDHS data.

#### Hurdle Binomial Regression Model

In the first model, we estimate ‘child’s years of schooling’ (which is a count variable) using a Hurdle regression model [31] which handles the demand for schooling as a function of two separate but related decisions. More specifically, it allows the decision to enrol to be treated as distinct from the decision to attain further years of schooling in one integrated model, while accounting for the right censoring issue that some children in the sample are continuing in school. We also tried to run a censored ordered probit model by separately entering children who had completed their entire schooling spells (uncensored observations) and children who had not completed their entire schooling spells (censored observations) into the likelihood function [32, 33]. However, our likelihood function did not converge and, as a result, we are not able to report results from the censored ordered probit estimation.

As noted above, 33.6 per cent of the children in the sample have no education or have never enrolled in school, hence ‘years of schooling’ has a large number of zeroes. In this case, treating count data on years of schooling as a continuous variable and estimating the linear regression model to examine the determinants of schooling may result in inefficient, inconsistent and biased estimates. Additionally, the zeros in the ‘years of schooling variable’ are generated by the decision to enrol a child in school or not. It is potentially a different process from that of positive counts, which are the positive number of years a child continues in school conditional on enrolment. Thus, zero (enrolment in school) is a “hurdle” that the child must clear before reaching positive counts. Hurdle regression models combine a binary model (e.g., logit) to predict zeros and a zero-truncated Poisson or zero-truncated negative binomial model to predict nonzero counts. However, in the Poisson Regression Model, the probability of a count is determined by a Poisson distribution in which the mean of the distribution is a function of the independent variables and the conditional mean of the outcome is assumed to be equal to the conditional variance [34–36]. This assumption does not fit into our outcome variable ‘years of schooling’ because the conditional variance exceeds the conditional mean resulting in *overdispersion*. The *Negative Binomial Regression Model* (NBRM) addresses this issue by allowing the variance to exceed the mean and produces more efficient estimators [34–38]. Thus, in the second stage, we have estimated a zero-truncated negative binomial model to predict nonzero counts.

Following Saffari et al. (2012) [39], we consider a hurdle negative binomial logit regression model in which the response variable years of schooling *Yi* (*i* = 1… *n*) has the following probability distribution and *Yi* = 0 is observed with a significantly higher frequency,

$$Pr\left(Y_{i}=y_{i}\right)=\{\begin{array}{l} \;w_{0},\;y_{i}=0,\\ \left(1-w_{0}\right)\frac{\Gamma \left(y_{i}+\alpha^{-1}\right)}{\Gamma \left(yi+1\right)\Gamma \left(\alpha^{-1}\right)}\;\frac{{\left(1+\alpha \mu_{i}\right)}^{-\alpha^{\begin{array}{c} -1 \end{array}}-y_{i}}\alpha^{y_{i}}\mu_{i}^{y_{i}}}{1-\left(1+\alpha \mu i\right)-a^{-1}},\;y_{i}>0, \end{array}$$ (1)

or

$$Pr\left(Yi=yi\right)=\{\begin{array}{l} \;w_{0},\;y_{i}=0,\\ \left(1-w_{0}\right)\frac{g}{1-\left(1+\alpha \mu i\right)-a^{-1}},\;y_{i}>0, \end{array}$$ (2)

Where

$$g=\text{g}\left(y_{i}\;;\;\mu_{i},\;\alpha \right)=\frac{\Gamma \left(y_{i}+\alpha^{-1}\right)}{\Gamma \left(yi+1\right)\Gamma \left(\alpha^{-1}\right)}{\left(1+\alpha \mu_{i}\right)}^{-\alpha^{\begin{array}{c} -1 \end{array}}-y_{i}}\alpha^{y_{i}}\mu_{i}^{y_{i}}$$ (3)

where *α* (≥ 0) is a dispersion parameter that is assumed not to depend on covariates. In addition, we suppose 0 < *w*_0_ < 1 and *w*_0_ = *w*_0_(*z*_*i*_) satisfy

$$logit\left(w_{0}\right)=\log\left(\frac{w_{0}}{1-w_{0}}\right)=\overset{m}{\underset{j=1}{\sum }}z_{ij}\delta_{j}$$ (4)

where (*z*_*i*1_ = 1, *z*_*i*2_,…, *z*_*im*_) is the *i*-th row of covariate matrix *Z* and (*δ*_1_, *δ*_2_,…, *δ*_*m*_) is an unknown *m*-dimensional column vector of parameters. Here, the nonnegative function *w*_0_ is modelled via logit link function.

The NBRM addresses the variation due to unobserved heterogeneity [36]. For a given combination of independent variables (y_i_s) there is a distribution of mean values (μs) rather than a single μ. The conditional mean is still μ, but the variance will be greater because of the error term. In other words unobserved heterogeneity leads to overdisperson, which invalidates the assumption underlying the Poisson model but NBRM addresses this issue [40].

To control for right censoring and exposure differences between children of different ages the Hurdle model controls for exposure time in the second stage *Negative Binomial regression*. A link function measuring exposure was included:

$$School\;Exposure\;time=Child^{\prime }s\;age-7$$ (5)

(note that the official age for starting primary school in Ethiopia is age 7)

For each child in the sample the model estimated their years of schooling allowing for different exposure time by accounting for the fact that some children in the sample are currently continuing in school.

The Hurdle negative binomial model, which is by far the most popular hurdle model in practice, ensures identification based on functional form [41]. Other studies in the literature have also estimated this model on a count data with many zeros (for example, [39, 42, 43]).

#### Ordered Probit Model

To examine age-grade distortion, we estimate an ordered probit regression model given the natural ordering of age-for-grade. The ordered probit model is based on a latent regression of the form

$$y_{i}{}^{*}=X_{i}{}^{\prime }\beta +\varepsilon_{i}$$ (6)

Where y* is an unobservable latent variable, *β* is a vector of parameter estimates and *X* of different household characteristics, and *ε* is an error term normally distributed with zero mean and variance one [44].

The response variable for schooling outcomes, *age-for-grade*, is an ordered variable taking on values 0, 1 and 2. Based on self-reported answers to the question of whether or not the child has ever been to school, *age-for-grade* equals 0 if a child has never attended school. For those children who have had some schooling, we follow Patrinos and Psacharopoulos (1997) [29] and define a schooling-for-age variable as follows:

$$schooling-for-age=100*\left[\frac{years\;of\;schooling}{age-7}\right]$$ (7)

Accordingly, all dispersions in age are measured from the age of 7-years old, which is the official age for starting primary school in Ethiopia. A measure of 100 indicates complete schooling attainment, and a value greater than 0 but lower than 100 indicates falling behind.

Some of the children in the sample have a value greater than 100 for the schooling-for-age variable, which is possible if the child has been to some form of pre-school. Hence, *age-for-grade* equals 1 if the child has a schooling value lower than 100 but greater than 0, and equals 2 if the child has a schooling value of 100 or above. This measure of schooling outcomes takes into account all the available information on school attendance and dropping out, and gives us an indication of those children who may have fallen behind in their schooling attainment.

### Explanatory variables

The relationship between maternal autonomy and child schooling has been widely discussed in the literature. Studies by Williams (1990) [45] and Thomas et al. (1997) [46] examine how maternal control over financial resources improves child-related outcomes. Women’s autonomy is typically defined as their ability to influence decisions about themselves or close household members through control over material resources (including food, income, land and other forms of wealth) and social resources (including knowledge, power and prestige) within the family, community and society at large [12,47,48]. In the rural Ethiopian context, there were some studies that looked at the position of women in the household vis-à-vis divorce settlement laws and assets brought to marriage in an intrahousehold bargaining framework [49–51]. Women’s autonomy is a multidimensional concept that aims to measure not only women’s ability to control resources but also their ability to choose and control different outcomes and enhance their self-esteem [52, 53].

A key advantage of the EDHS dataset is that it contains a wide range of questions that assess maternal decision-making autonomy on a range of issues. Our analysis uses direct indicators of maternal autonomy in the following dimensions: mothers’ participation in household decision making, freedom of movement, independent access to resources, and access to new liberating ideas [54]. These indicators are obtained from mothers’ self-reported answers to the following questions in the EDHS: (*i*) Is the mother involved in decisions concerning her own health?; (*ii*) Does the mother have ownership rights over the house/land?; (*iii*) Does the mother need permission to visit family/relatives?; (*iv*) Does the mother believe in family planning (Birth spacing)?; (*v*) Does the mother know about the law prohibiting wife-beating? Malhotra et al. (2002) [55], Smith (2003) [56] and Basu and Stephenson (2005) [57] note the appropriateness of considering different dimensions of autonomy given the multidimensional nature of the concept and the possibility that different dimensions may affect the variable of interest, such as health or education, differently. We have checked for the correlation between the five indicators of the mother's autonomy using a Wald Test for independence of variables and results indicate that all five variables are significantly independent of each other. In addition, we tried to create an index (weighting all the measures equally) over the five autonomy variables and computed an average effect of these measures. However, including this index in our models did not improve the model specifications, and the likelihood function also did not come up as significant. Consequently, we decided to use the 5 autonomy variables independently in our models.

The other explanatory variables used in this paper include a range of control variables that have been shown to affect schooling in the wider literature. These variables include the household’s demographic, socio-economic and labour market characteristics. We also control for gender of the child to identify potential gender-disparities in schooling outcomes. Dancer and Rammohan (2009) [58] note that gender-disparities in educational outcomes are widely observed in developing countries, with girls’ educational attainment being lower than boys’. We also control for parental characteristics such as age and educational status. Parental age indicates parents’ experience and knowledge, which affects their ability to make schooling decisions [28]. Apart from the direct intergenerational effects, parental education may change the bargaining power in the household, with educated mothers directing more resources towards children’s schooling [59]. Moreover, there is evidence pointing to differential effects of paternal and maternal education on children’s schooling (for example, [60]).

We also control for a number of household characteristics that have been shown to play a significant role in determining various child outcomes. These include household size, number of siblings under five years of age, and household wealth [7, 61, 62]. To control for wealth, we use a wealth index, which divides households into five different wealth quintiles (WEALTH1-WEALTH5). The lowest quintile, WEALTH1, represents the poorest 20 per cent of households and the highest quintile, WEALTH5, the wealthiest 20 per cent. We also include a dummy for gender of the head of household. There is evidence indicating that ‘…both boys and girls living in female headed households show universally better school outcomes than children living in male-headed households when households with similar resources are compared’ [63]. This might reflect the tendency of women to direct a greater share of household expenditure to their children if they are able to control a larger share of household resources.

We also control for the mother’s ethnicity by including dummies for language family (of the first language spoken by the mother) to account for ethno-cultural differences and the influence of informal social institutions and practices in Ethiopia. Cultural differences among ethnic groups (in a multi-ethnic society like Ethiopia) may affect the household’s attitude towards child schooling and allocation of resources to children. The EDHS data do not provide information on the ethnic affiliation of households. In the absence of such information, we use language family to account for ethno-cultural differences. Language family better captures ethno-cultural differences than do regional dummies because people belonging to different ethnic groups may reside in the same region. According to the latest census, the most widely spoken language in Ethiopia is Afan Oromo with around 25 million people speaking the language, followed by Amharic at 21.6 million, Somali at 4.6 million and Tigrignya at 4.3 million [64]. In our regression, we have created dummies for Amharic, Somali, Tigrignya and the residual category ‘other languages’ with Afan Oromo taken as the base category.

### Descriptive Statistics

The summary statistics of variables is presented in Table 1. By and large, the summary statistics by gender of children mirrors the overall pattern. The proportion of children in the sample who had ever been enrolled is 66 per cent while the average years of schooling is 2.4 years. The average age of children is around 12-years old with boys accounting for 52 per cent of the sample. The proportion of children in the sample who are in the 8–14 age category (i.e. primary school age) is approximately 76 per cent.

**Table 1:** Summary statistics of variables.

Variables	Mean Total	Mean Male	Mean Female
**Dependent variables**			
Enrolment Status (= 1 if ever enrolled; 0 otherwise)	0.66	0.66	0.65
	(0.48)	(0.47)	(0.48)
Years of schooling (for those children ever enrolled)	2.42	2.41	2.42
	(2.61)	(2.59)	(2.64)
**Explanatory variables**			
***Child Characteristics***			
Age	12.30	12.33	12.14
	(3.38)	(3.41)	(3.29)
Gender of child (= 1 if male, 0 otherwise)	0.52		
	(0.50)		
***Household Characteristics***			
Household size	7.06	7.03	7.08
	(1.80)	(1.80)	(1.79)
No. Siblings 5-years old or younger	1.16	1.13	1.20
	(0.94)	(0.93)	(0.95)
Gender of Household head (= 1 if Male)	0.92	0.92	0.91
	(0.28)	(0.27)	(0.29)
Orthodox	0.43	0.41	0.44
	(0.49)	(0.49)	(0.50)
Muslim	0.31	0.32	0.30
	(0.46)	(0.46)	(0.46)
Catholic	0.01	0.01	0.01
	(0.12)	(0.12)	(0.12)
Protestant	0.24	0.24	0.23
	(0.43)	(0.43)	(0.42)
Other religion	0.02	0.01	0.02
	(0.12)	(0.12)	(0.13)
***Parental Characteristics***			
Father’s age	45.63	46.00	45.22
	(9.89)	(10.06)	(9.68)
Mother’s age	36.63	36.88	36.37
	(6.32)	(6.35)	(6.28)
Father’s education-none	0.55	0.56	0.54
	(0.50)	(0.50)	(0.50)
Father’s education- primary	0.38	0.37	0.39
	(0.49)	(0.48)	(0.49)
Fath. Edu.-secondary & above	0.07	0.07	0.07
	(0.26)	(0.26)	(0.26)
Mother’s education-none	0.76	0.76	0.75
	(0.43)	(0.43)	(0.43)
Mother’s education-primary	0.21	0.21	0.22
	(0.41)	(0.41)	(0.41)
Moth.edu -secondary & above	0.03	0.03	0.03
	(0.17)	(0.26)	(0.18)
***Autonomy Variables***			
Mother decides on her health care	0.75	0.75	0.75
	(0.43)	(0.43)	(0.44)
Mother needs permission to visit relatives	0.22	0.21	0.22
	(0.41)	(0.41)	(0.42)
Mother alone or jointly owns house/land	0.93	0.93	0.92
	(0.26)	(0.26)	(0.26)
Mother believes in family planning	0.46	0.45	0.46
	(0.50)	(0.50)	(0.50)
Mother knows there is a law against wife beating in Ethiopia	0.48	0.48	0.48
	(0.50)	(0.50)	(0.50)
***Location***			
Urban	0.14	0.14	0.14
	(0.34)	(0.34)	(0.35)
***Language of mother***			
Afan Oromo	0.34	0.34	0.34
	(0.47)	(0.47)	(0.47)
Amharic	0.30	0.30	0.30
	(0.46)	(0.46)	(0.46)
Tigrignya	0.06	0.06	0.06
	(0.24)	(0.26)	(0.24)
Somali	0.02	0.02	0.02
	(0.15)	(0.15)	(0.15)
Other	0.27	0.27	0.28
	(0.44)	(0.44)	(0.45)

The average household size in the sample is around 7 while the average number of siblings 5-years old or younger is 1. Table 1 also shows that over 90 per cent of household heads are male. In terms of religious affiliation, over 40 per cent are Orthodox (Coptic) Christians, Muslims account for 30 per cent and Protestants for 24 per cent.

We see a significant difference in the educational attainment of fathers and mothers: the proportion of mothers with no education is 76 per cent compared to 55 per cent for fathers. Similarly, 38 per cent of fathers had completed their primary education while the corresponding figure for mothers is 21 per cent.

Turning to variables measuring maternal autonomy, the summary statistics reveal that 75 per cent of mothers in the sample can decide (or are involved in decisions) about their health care. Moreover, less than a quarter of mothers must ask for permission to visit relatives. Nevertheless, 93 per cent of mothers report being a sole/joint owner of their house/land. However, close to 50 per cent of sampled mothers know there is a law that prohibits wife beating in Ethiopia. The proportion believing in family planning is even smaller, at 46 per cent.

Furthermore, only 14 per cent of households are located in urban areas, reflecting the fact that Ethiopia is predominantly an agrarian society. The language group variables included for the purpose of capturing ethno-cultural differences indicate that 34 per cent of children in the sample have mothers who speak Afan Oromo, followed by Amharic (the official language of Ethiopia) at 30 per cent.

### Ethics statement

The present analysis used only de-identified existing survey data collected by MEASURE DHS. The survey included in this study was approved by the Institutional Review Board of Macro International (home of MEASURE DHS) in Calverton in the United States of America and by the National Ethical Review Committees in the surveyed country. We obtained the raw survey data from MEASURE DHS which also gave consent for us to use the data for analysis.

## III. Discussion of Results

### Hurdle Negative Binomial model estimation

The main results of our analysis are presented in Tables 2 and 3. The results from the Hurdle model estimation for the total sample and by gender of children are presented in Table 2. The first equation of the Hurdle model determines whether a child is ever enrolled and the second determines positive years of schooling conditional on a child being ever enrolled. We fit a logit model at the enrolment stage and a negative binomial model to estimate years of schooling at the second stage. We are reporting in the table the Incidence Rate Ratio (IRR), which measures the percentage increase or decrease in the dependent variable for a unit change in the independent variable (or a discrete change from 0 to 1 for a dummy variable). The IRR is commonly reported when estimating count data models [65]. The exact percentage change is determined by how far above or below the IRR is from 1.

**Table 2:** Hurdle Negative Binomial model estimation results.

	Total		Male		Female	
Variables	IRR Logit	IRR Neg. Binomial	IRR Logit	IRR Neg. Binomial	IRR Logit	IRR Neg. Binomial
***Child Characteristics***						
Gender (= 1 if the child is male)	0.949	0.990				
	(0.107)	(0.069)				
***Household Characteristics***						
Household size	0.945	0.921***	0.863**	0.923**	1.037	0.910**
	(0.041)	(0.025)	(0.863)	(0.033)	(0.065)	(0.035)
Number of siblings aged 5 or younger	0.917	1.082	1.164	1.146*	0.713***	1.025
	(0.074)	(0.057)	(0.132)	(0.081)	(0.082)	(0.079)
Gender of Household head (= 1 if male)	1.112	1.206	1.411	1.170	0.844	1.183
	(0.224)	(0.139)	(0.412)	(0.195)	(0.240)	(0.192)
Religion (= 1 if Orthodox)	Reference	Reference	Reference	Reference	Reference	Reference
Religion (= 1 if Muslim)	1.056	0.887	1.335	0.857	0.849	0.938
	(0.176)	(0.086)	(0.305)	(0.119)	(0.206)	(0.129)
Religion (= 1 if Catholic)	2.099	0.739	3.045*	0.539*	1.409	1.026
	(1.100)	(0.197)	(2.026)	(0.202)	(1.157)	(0.360)
Religion (= 1 if Protestant)	1.395*	0.959	2.071***	0.922	0.873	0.993
	(0.254)	(0.104)	(0.513)	(0.135)	(0.234)	(0.157)
Religion (= 1 if Other)	0.639	0.823	1.800	1.161	0.186*	0.318*
	(0.330)	(0.301)	(1.062)	(0.476)	(0.165)	(0.201)
Wealth Index (= 1 if household belongs to 1st quintile)	Reference	Reference	Reference	Reference	Reference	Reference
					
Wealth Index (= 1 if household belongs to 2nd quintile)	1.624***	1.144	1.623*	1.171	1.648*	1.143
	(0.293)	(0.153)	(0.402)	(0.215)	(0.430)	(0.210)
Wealth Index (= 1 if household belongs to 3rd quintile)	2.508***	1.093	2.235***	1.235	2.853***	0.984
	(0.446)	(0.137)	(0.546)	(0.218)	(0.744)	(0.167)
Wealth Index (= 1 if household belongs to 4th quintile)	3.912***	1.346**	3.813***	1.271	4.053***	1.422**
	(0.705)	(0.167)	(0.959)	(0.219)	(1.070)	(0.240)
Wealth Index (= 1 if household belongs to 5^th^ quintile)	6.124***	1.506**	6.078***	1.366	6.202***	1.747**
	(1.440)	(0.238)	(1.899)	(0.296)	(2.268)	(0.398)
***Parental Characteristics***						
Father's Age	0.983**	1.000	0.969**	1.001	0.999	1.000
	(0.008)	(0.005)	(0.012)	(0.007)	(0.012)	(0.008)
Father’s education (= 1 if no education)	Reference	Reference	Reference	Reference	Reference	Reference
Father's education (= 1 if primary school)	1.786***	1.010	1.853***	1.168	1.856***	0.834
	(0.228)	(0.084)	(0.324)	(0.136)	(0.350)	(0.099)
Father's education (= 1 if secondary or above)	4.008***	1.028	4.170***	1.000	4.936***	0.929
	(1.235)	(0.141)	(1.528)	(0.174)	(2.482)	(0.193)
Mother's age	0.938***	0.951***	0.964*	0.953***	0.917***	0.946***
	(0.013)	(0.008)	(0.019)	(0.011)	(0.018)	(0.011)
Mother’s education (= 1 if no education)	Reference	Reference	Reference	Reference	Reference	Reference
Mother’s education (= 1 if primary school)	1.264	0.999	1.006	1.039	1.580**	0.962
	(0.185)	(0.083)	(0.203)	(0.126)	(0.339)	(0.110)
Mother’s education (= 1 if secondary or above)	0.954	1.107	2.362	1.292	0.498	1.019
	(0.413)	(0.175)	(1.401)	(0.277)	(0.279)	(0.230)
***Autonomy Variables***						
= 1 if the mother decides/is involved in decisions on her health care	1.194	1.348***	1.398	1.426**	0.970	1.233
	(0.175)	(0.134)	(0.296)	(0.205)	(0.199)	(0.171)
= 1 if the mother needs permission for visits to family/relatives	0.483***	1.168	0.474***	0.900	0.495***	1.486***
	(0.079)	(0.125)	(0.112)	(0.133)	(0.115)	(0.224)
= 1 if the mother alone/jointly owns a house/land	1.679***	1.220*	1.354	1.246	2.180**	1.158
	(0.373)	(0.147)	(0.422)	(0.222)	(0.678)	(0.184)
= 1 mother thinks it is wise to have a balanced family life- birth spacing of children	1.202	1.014	1.278	1.028	1.129	1.017
	(0.148)	(0.081)	(0.220)	(0.116)	(0.200)	(0.111)
= 1 if mother knows there is a law in Ethiopia that prohibits a husband from beating	1.098	1.014	0.954	1.020	1.289	0.997
	(0.129)	(0.076)	(0.156)	(0.104)	(0.220)	(0.105)
***Location***						
= 1 if the place of residence is urban	2.448***	1.400**	2.955***	1.715**	2.110**	1.078
	(0.585)	(0.219)	(1.025)	(0.372)	(0.741)	(0.213)
***Language of mother***						
Language (= 1 if Afan Oromo)	Reference	Reference	Reference	Reference	Reference	Reference
Language (= 1 if Amharic)	1.537**	1.220*	1.075	1.156	2.332***	1.232
	(0.271)	(0.125)	(0.260)	(0.165)	(0.605)	(0.179)
Language (= 1 if Tigringya)	4.976***	1.730***	3.446***	1.624***	8.007***	1.864
	(1.058)	(0.199)	(1.054)	(0.264)	(2.421)	(0.303)
Language (= 1 if Somali)	0.677	1.427	1.171	1.439	0.321***	1.292
	(0.182)	(0.386)	(0.402)	(0.464)	(0.137)	(0.508)
Language (= 1 if Other)	0.790	1.100	0.742	1.116	0.873	1.114
	(0.124)	(0.111)	(0.157)	(0.147)	(0.205)	(0.163)
_constant	0.126***	0.000	0.105***	0.000***	0.108**	0.000
	(0.066)	(0.000)	(0.077)	(0.000)	(0.081)	(0.000)
Number of Observations	11949		6217		5732	
Wald chi2 (28)	594.69		328.75		342.1	
Log pseudolikelihood	-29360.04		-15149.39		14065.73	

Note: Robust standard errors in parentheses

*** p<0.01

** p<0.05

* p<0.1.

Our results confirm that the mother’s economic and social autonomy in the household are significant determinants of schooling outcomes for children. As shown in Table 2, a child’s likelihood of ever being enrolled significantly increases if the mother owns a house/land alone/jointly. The effect of this autonomy indicator, however, is significant only for girls. In terms of magnitude, having a mother who owns a house/land increases the likelihood of enrolment for girls by 118 per cent compared to those whose mothers don’t own such property. This might be a result of economically empowered mothers allocating more resources towards their daughters. There is literature indicating that the relative bargaining power between parents in the family may result in different decisions regarding investment in their children. Thomas (1990, 1994) [66, 67], for example, find evidence that mothers show preference to daughters while fathers prefer sons in their allocation of resources. Luz and Agadjanina (2015) [68] also conclude that mothers bargaining power is important for education of daughters using data from rural Mozambique.

Women’s participation in decision making on their own health care is considered an important autonomy variable to analyse women’s empowerment at the household level [69]. Autonomy that supports health care decision-making is associated with better health outcomes for women [70]. Our results show that autonomy in health care decisions is a statistically significant determinant at the second stage, but only for boys. Having a mother with such autonomy increases the expected years of schooling for boys by 43 per cent.

The other significant mother’s autonomy indicator is whether the mother needs permission to visit relatives. Having a mother that requires permission reduces the likelihood of enrolment by around 52 per cent for both boys and girls. This variable is also significant for how long girls stay in school but with unexpected sign. We cross tabulated this variable with other regressors in our model and found out that this result was based on small observations, which may have affected the estimate. This variable represents the mother’s freedom of movement, her social status and the respect she carries in the household. If she does need permission to visit relatives, then she has less bargaining power in the household to influence the resources allocated for the schooling of her children. Additionally, maternal freedom to leave the house and visit relatives (or friends for that matter) may create the opportunity to exchange ideas and information to improve her children’s well-being, including their schooling outcomes. Therefore, this result may also be an indication that a mother who requires permission to leave the house may not have as much opportunity to interact with family (and friends) as a mother who does not require such permission [71].

Turning to other explanatory variables included in the model, household size significantly decreases the average years of schooling. An increase in household size by 1 results in the average years of schooling decreasing by approximately 8 per cent. This variable is also significant at the enrolment stage for boys, decreasing the likelihood of enrolment by 14 per cent. This may reflect the possibility that households with more members may face different constraints (including resource and time), which may adversely affect schooling outcomes for children. It is also apparent from the results that having more siblings aged five or under is only significant for the enrolment of girls. An additional sibling under 5 years of age reduces girls likelihood of enrolment by 28 per cent. This result might be capturing the increased opportunity cost of schooling in cases where older children (especially girls) are expected to assist in looking after their younger siblings. Similarly, Cockburn and Dostie (2007) [72] found that the number of infants in the household reduced the likelihood that a child would attend school in rural Ethiopia.

Overall, paternal education is a more significant determinant than maternal education: children whose fathers had attained some level of education were more likely to enrol compared to those whose fathers had no education. Maternal education at the primary level is also significant but only for girls. Daughters whose mothers had completed primary school are 58 per cent more likely to enrol than those whose mothers had no education. However, parental education is not a significant determinant of how long children will remain in school. Parental age is also shown to be a significant determinant. Father’s age is significant only for boys with a one year increase in father’s age reducing the likelihood that boys would enrol by 3 per cent. Mother’s age is significant for both sexes and increase in mother’s age reduces the probability of enrolment as well as expected years of schooling.

Consistent with the literature, our findings indicate that an increase in household wealth significantly increases the probability of a child being enrolled in school [61, 73–76]. The probability of enrolment for children coming from the highest wealth quintile is about 512 per cent higher than that of children from the bottom quintile. But the effect of wealth on expected years of schooling is only significant for girls from highest two quintiles. For example, the average years of schooling for girls coming from a household that belongs to the 5^th^ quintile is 75 per cent more than girls coming from the bottom quintile.

The urban-rural dummy shows a highly significant and positive association between urban residence and school enrolment for both sexes. However, it is significant for increasing average years of schooling only for boys. Children in urban areas have better schooling outcomes because rural children are more likely to engage in jobs that conflict with human capital accumulation compared to their urban counterparts [77]. Cockburn and Dostie (2007) [72] note that children in rural Ethiopia are involved in household farm or domestic work activities such as fetching wood and water and herding and that child labour is cited as the main reason for school non-attendance.

Finally, the language group dummies indicate that children from households whose mothers speak Tigrigna as a first language are more likely to enrol, as well as stay in school longer, compared to the base category of Afan Oromo speakers. This result may be capturing differences in access to schools. Demand side factors may also be responsible, as Afan Oromo speakers are settled on fertile agricultural land that produces a surplus in Ethiopia. As a result, children may be required to assist in farming activities whereas there would be no such demand on children’s time in areas where agricultural opportunities are lower. The results further show that girls whose mothers first language is Amharic are more likely to enrol than those whose mothers first language is Afan Oromo, but this result is not significant for boys. Somali speakers are less likely to be enrolled compared to the Oromo. This may reflect that the predominantly nomadic lifestyle of the Somali speakers adversely affects the schooling outcomes of their children.

### Ordered Probit Model

We have estimated an ordered probit regression model to capture age appropriate grade achievement with *age-for-grade* as a dependent variable. As noted earlier, the dependent variable, *age-for-grade*, consists of three ordered choices: 0 for a child who has never attended school; 1 for a child who is lagging behind the correct grade for age; and 2 for a child who is in the correct grade for age. Mani et al. (2013) have used similar measures of relative grade attainment to analyse longer-term schooling progression for rural Ethiopia. Marginal effects from our estimation are presented in Table 3 below. We will focus our discussion on the marginal effects from the overall sample where there isn’t significant difference between boys and girls.

**Table 3:** Marginal effects from ordered probit estimation.

Variables	Marginal Effects Total			Marginal Effects Male			Marginal Effects Female		
	Never enrolled	Wrong grade for age	No age grade distortion	Never enrolled	Wrong grade for age	No age grade distortion	Never enrolled	Wrong grade for age	No age grade distortion
***Child Characteristics***									
Gender (= 1 if the child is male)	-0.0245***	0.0076***	0.0169***						
	(0.0069)	(0.0022)	(0.0048)						
***Household Characteristics***									
Household size	-0.0046**	0.0014**	0.0032**	-0.0052	0.0015	0.0037	-0.0036	0.0012	0.0024
	(0.0023)	(0.0007)	(0.0016)	(0.0032)	(0.0010)	(0.0023)	(0.0032)	(0.0011)	(0.0022)
The number of siblings aged 5 or younger	0.0228***	-0.0071***	-0.0157***	0.0217***	-0.0064***	-0.0153***	0.0231***	-0.0076***	-0.0156***
	(0.0048)	(0.0015)	(0.0033)	(0.0067)	(0.0020)	(0.0047)	(0.0069)	(0.0023)	(0.0047)
Gender of Household head (= 1 if male)	0.0105	-0.0033	-0.0073	0.0160	-0.0047	-0.0113	0.0033	-0.0011	-0.0022
	(0.0120)	(0.0037)	(0.0083)	(0.0169)	(0.0050)	(0.0119)	(0.0170)	(0.0056)	(0.0115)
Religion (= 1 if Orthodox)	Reference	Reference	Reference	Reference	Reference	Reference	Reference	Reference	Reference
Religion (= 1 if Muslim)	0.0068	-0.0021	-0.0047	-0.0179	0.0053	0.0126	0.0329**	-0.0107**	-0.0222**
	(0.0105)	(0.0033)	(0.0072)	(0.0145)	(0.0043)	(0.0102)	(0.0151)	(0.0049)	(0.0102)
Religion (= 1 if Catholic)	-0.0330	0.0103	0.0227	-0.0340	0.0100	0.0240	-0.0371	0.0121	0.0250
	(0.0305)	(0.0095)	(0.0210)	(0.0439)	(0.0130)	(0.0309)	(0.0419)	(0.0137)	(0.0282)
Religion (= 1 if Protestant)	-0.0044	0.0014	0.0030	-0.0252	0.0074	0.0177	0.0189	-0.0062	-0.0127
	(0.0125)	(0.0039)	(0.0086)	(0.0174)	(0.0052)	(0.0123)	(0.0178)	(0.0058)	(0.0120)
Religion (= 1 if Other)	0.0141	-0.0044	-0.0097	-0.0536	0.0158	0.0378	0.0853**	-0.0278**	-0.0574**
	(0.0291)	(0.0091)	(0.0200)	(0.0392)	(0.0116)	(0.0276)	(0.0432)	(0.0141)	(0.0291)
Wealth Index (= 1 if household belongs to 1^st^ quintile)	Reference	Reference	Reference	Reference	Reference	Reference	Reference	Reference	Reference
Wealth Index (= 1 if household belongs to 2nd quintile)	-0.0481***	0.0150***	0.0331***	-0.0541***	0.0160***	0.0381***	-0.0441***	0.0144***	0.0297***
	(0.0109)	(0.0034)	(0.0075)	(0.0150)	(0.0045)	(0.0106)	(0.0157)	(0.0051)	(0.0106)
Wealth Index (= 1 if household belongs to 3^rd^ quintile)	-0.0802***	0.0250***	0.0552***	-0.0923***	0.0273***	0.0651***	-0.0681***	0.0223***	0.0459***
	(0.0108)	(0.0034)	(0.0075)	(0.0150)	(0.0046)	(0.0107)	(0.0155)	(0.0051)	(0.0105)
Wealth Index (= 1 if household belongs to 4^th^ quintile)	-0.1344***	0.0418***	0.0926***	-0.1477***	0.0436***	0.1041***	-0.1233***	0.0403***	0.0830***
	(0.0110)	(0.0036)	(0.0077)	(0.0152)	(0.0048)	(0.0110)	(0.0157)	(0.0053)	(0.0108)
Wealth Index (= 1 if household belongs to 5^th^ quintile)	-0.2265***	0.0705***	0.1560***	-0.2431***	0.0718***	0.1714***	-0.2043***	0.0667***	0.1376***
	(0.0152)	(0.0053)	(0.0107)	(0.0205)	(0.0071)	(0.0147)	(0.0226)	(0.0079)	(0.0154)
***Parental Characteristics***									
Father's Age	-0.0002	0.0001	0.0001	0.0004	-0.0001	-0.0003	-0.0009	0.0003	0.0006
	(0.0005)	(0.0001)	(0.0003)	(0.0007)	(0.0002)	(0.0005)	(0.0007)	(0.0002)	(0.0004)
Father’s education (= 1 if no education)	Reference	Reference	Reference	Reference	Reference	Reference	Reference	Reference	Reference
Father’s education (= 1 if primary school)	-0.0622***	0.0194***	0.0429***	-0.0658***	0.0194***	0.0463***	-0.0612***	0.0200***	0.0412***
	(0.0084)	(0.0027)	(0.0058)	(0.0117)	(0.0036)	(0.0083)	(0.0121)	(0.0041)	(0.0082)
Father’s education (= 1 if secondary or above)	-0.1098***	0.0342***	0.0757***	-0.1320***	0.0390***	0.0931***	-0.0948***	0.0310***	0.0638***
	(0.0164)	(0.0053)	(0.0113)	(0.0232)	(0.0072)	(0.0163)	(0.0232)	(0.0077)	(0.0156)
Mother's age	-0.0001	0.0000	0.0001	-0.0012	0.0004	0.0009	0.0011	-0.0004	-0.0007
	(0.0008)	(0.0003)	(0.0006)	(0.0011)	(0.0003)	(0.0008)	(0.0011)	(0.0004)	(0.0008)
Mother’s education (= 1 if no education)	Reference	Reference	Reference	Reference	Reference	Reference	Reference	Reference	Reference
Mother’s education (= 1 if primary school)	-0.0329***	0.0103***	0.0227***	-0.0154	0.0045	0.0108	-0.0477***	0.0156***	0.0321***
	(0.0097)	(0.0030)	(0.0067)	(0.0136)	(0.0040)	(0.0096)	(0.0136)	(0.0045)	(0.0091)
Mother’s education (= 1 if secondary or above)	-0.1790***	0.0557***	0.1233***	-0.1537***	0.0454***	0.1083***	-0.1949***	0.0636***	0.1312***
	(0.0247)	(0.0082)	(0.0168)	(0.0350)	(0.0109)	(0.0244)	(0.0348)	(0.0122)	(0.0231)
***Autonomy variables***									
= 1 if the mother decides/involved in the decision on her health care	-0.0216**	0.0067**	0.0149**	-0.0228*	0.0067*	0.0161*	-0.0247*	0.0081*	0.0167*
	(0.0091)	(0.0028)	(0.0063)	(0.0124)	(0.0037)	(0.0088)	(0.0133)	(0.0043)	(0.0090)
= 1 if the mother needs permission for visits to family/relatives	0.0363***	-0.0113***	-0.0250***	0.0341***	-0.0101***	-0.0241***	0.0368***	-0.0120***	-0.0248***
	(0.0094)	(0.0030)	(0.0065)	(0.0129)	(0.0038)	(0.0091)	(0.0137)	(0.0045)	(0.0092)
= 1 if the mother alone/jointly owns a house/land	-0.0026	0.0008	0.0018	0.0226	-0.0067	-0.0159	-0.0291	0.0095	0.0196
	(0.0126)	(0.0039)	(0.0087)	(0.0177)	(0.0052)	(0.0125)	(0.0179)	(0.0058)	(0.0120)
= 1 mother thinks it is wise to have a balanced family life- birth spacing of children	-0.0334***	0.0104***	0.0230***	-0.0239**	0.0071**	0.0168**	-0.0415***	0.0136***	0.0280***
	(0.0078)	(0.0025)	(0.0054)	(0.0107)	(0.0032)	(0.0076)	(0.0114)	(0.0038)	(0.0077)
= 1 if mother knows there is law in Ethiopia that prohibits a husband from beating wife	-0.0193***	0.0060***	0.0133***	-0.0035	0.0010	0.0025	-0.0353***	0.0115***	0.0238***
	(0.0073)	(0.0023)	(0.0050)	(0.0101)	(0.0030)	(0.0071)	(0.0105)	(0.0035)	(0.0071)
***Location***									
= 1 if the place of residence is urban	-0.0768***	0.0239***	0.0529***	-0.0608***	0.0179***	0.0428***	-0.1009***	0.0330***	0.0680***
	(0.0143)	(0.0045)	(0.0098)	(0.0198)	(0.0059)	(0.0140)	(0.0205)	(0.0069)	(0.0138)
***Language of mother***									
Language (= 1 if Afan Oromo)	Reference	Reference	Reference	Reference	Reference	Reference	Reference	Reference	Reference
Language (= 1 if Amharic)	-0.0636***	0.0198***	0.0438***	-0.0363**	0.0107**	0.0256**	-0.0911***	0.0298***	0.0614***
	(0.0118)	(0.0037)	(0.0081)	(0.0161)	(0.0048)	(0.0114)	(0.0170)	(0.0058)	(0.0115)
Language (= 1 if Tigringya)	-0.1679***	0.0523***	0.1156***	-0.1421***	0.0420***	0.1002***	-0.1930***	0.0630***	0.1300***
	(0.0148)	(0.0050)	(0.0102)	(0.0206)	(0.0065)	(0.0145)	(0.0211)	(0.0075)	(0.0142)
Language (= 1 if Somali)	0.0933***	-0.0290***	-0.0643***	0.0568***	-0.0168***	-0.0400***	0.1459***	-0.0476***	-0.0982***
	(0.0163)	(0.0051)	(0.0113)	(0.0216)	(0.0064)	(0.0152)	(0.0247)	(0.0082)	(0.0168)
Language (= 1 if other)	0.0080	-0.0025	-0.0055	0.0142	-0.0042	-0.0100	0.0012	-0.0004	-0.0008
	(0.0099)	(0.0031)	(0.0068)	(0.0138)	(0.0041)	(0.0097)	(0.0142)	(0.0046)	(0.0096)
Observations	11,949	11,949	11,949	6,217	6,217	6,217	5,732	5,732	5,732

Notes: Standard errors in parenthesis

*** p<0.01

** p<0.05

* p<0.1.

The results of this model further confirm that maternal autonomy indicators are significant determinants of relative grade attainment. The probability that a child has never attended school is around 2.2 per cent lower if the mother is involved in her own health care decisions. Having a mother with this form of independence in the household increases the likelihood that a child has attended school, with the probability of being in the correct grade for age rising by 1.6 per cent. We can also see that children whose mothers need permission to visit relatives are more likely to have never attended school. The probability that these children would attend the correct grade for their age is lower by 2.5 per cent. Similarly, mother’s view on family planning is also a significant predictor, which increases the likelihood that a child has been to school (whether attending the correct grade for their age or otherwise). Interestingly, mother’s knowledge of the existence of a law that prohibits wife beating is a significant determinant of age-grade-distortion for girls, but insignificant for boys. The probability that girls would lag behind the correct grade for their age falls by 3.5 per cent if their mothers know about this law. Presumably, mother’s knowledge of this law may reduce the incidence of domestic violence which may create a conducive environment to enhance daughters performance at school.

Furthermore, the ordered probit estimates reveal that parental educational attainment has a significant impact on children’s schooling progression. We can see that mothers primary school completion is only significant for girl’s performance. For instance, girls whose mothers’ had completed primary education have around 3 per cent higher chance of being in the correct grade for age. Father’s education and mother’s completion of secondary school is important for both boys and girls. By and large, other variables in the regression follow the overall pattern of the Hurdle model estimation.

## IV. Conclusion

This paper examines the effect of maternal autonomy on child schooling outcomes in Ethiopia. There is evidence from the wider literature that a mother’s socioeconomic status is positively associated with the well-being of her children. However, the role of maternal autonomy in child schooling has not been studied in the Ethiopian context. We use both direct and indirect indicators of mothers’ economic and social autonomy from the latest round of the Ethiopian Demographic and Health survey (2010–2011) to investigate the effects of maternal autonomy on child schooling (after controlling for several other variables that affect schooling outcomes). The empirical strategy uses a Hurdle Negative Binomial regression model to estimate years of schooling and an ordered probit model to examine age grade distortion in schooling progression.

The results show that maternal economic and social autonomy are significant determinants of child schooling outcomes in Ethiopia. Having an economically empowered mother who owns a house/land alone/jointly increases the likelihood of enrolment for daughters. A mother’s authority to decide on matters concerning her own health care is found to be significant for expected years of schooling for boys. Moreover, the probability of enrolment decreases if the mother requires permission to visit relatives. These outcomes, by and large, are mirrored in the ordered probit regression results confirming the significance of mother’s empowerment for children’s schooling progression. In addition, mothers belief in family planning comes out significant in the ordered probit estimation. We also find that mother’s knowledge of a law prohibiting wife beating is a significant determinant increasing the likelihood that girls would attend the correct grade for their age.
